# Identification of missing variants by combining multiple analytic pipelines

**DOI:** 10.1186/s12859-018-2151-0

**Published:** 2018-04-16

**Authors:** Yingxue Ren, Joseph S. Reddy, Cyril Pottier, Vivekananda Sarangi, Shulan Tian, Jason P. Sinnwell, Shannon K. McDonnell, Joanna M. Biernacka, Minerva M. Carrasquillo, Owen A. Ross, Nilüfer Ertekin-Taner, Rosa Rademakers, Matthew Hudson, Liudmila Sergeevna Mainzer, Yan W. Asmann

**Affiliations:** 1 Department of Health Sciences Research, Mayo Clinic, Jacksonville, FL 32224 USA; 2 Department of Neuroscience, Mayo Clinic, Jacksonville, FL 32224 USA; 3 Division of Biomedical Statistics and Informatics, Department of Health Sciences Research, Mayo Clinic, Rochester, MN 55905 USA; 4 Department of Clinical Genomics, Mayo Clinic, Jacksonville, FL 32224 USA; 5 Department of Neurology, Mayo Clinic, Jacksonville, FL 32224 USA; 6 National Center for Supercomputing Applications, University of Illinois at Urbana-Champaign, Urbana, IL 61801 USA; 7 Carl R Woese Institute for Genomic Biology, Carver Biotechnology Center and Department of Crop Sciences, University of Illinois at Urbana-Champaign, Urbana, IL 61801 USA

**Keywords:** Missing variants, Combining multiple bioinformatics pipelines, Rare variants

## Abstract

**Background:**

After decades of identifying risk factors using array-based genome-wide association studies (GWAS), genetic research of complex diseases has shifted to sequencing-based rare variants discovery. This requires large sample sizes for statistical power and has brought up questions about whether the current variant calling practices are adequate for large cohorts. It is well-known that there are discrepancies between variants called by different pipelines, and that using a single pipeline always misses true variants exclusively identifiable by other pipelines. Nonetheless, it is common practice today to call variants by one pipeline due to computational cost and assume that false negative calls are a small percent of total.

**Results:**

We analyzed 10,000 exomes from the Alzheimer’s Disease Sequencing Project (ADSP) using multiple analytic pipelines consisting of different read aligners and variant calling strategies. We compared variants identified by using two aligners in 50,100, 200, 500, 1000, and 1952 samples; and compared variants identified by adding single-sample genotyping to the default multi-sample joint genotyping in 50,100, 500, 2000, 5000 and 10,000 samples. We found that using a single pipeline missed increasing numbers of high-quality variants correlated with sample sizes. By combining two read aligners and two variant calling strategies, we rescued 30% of pass-QC variants at sample size of 2000, and 56% at 10,000 samples. The rescued variants had higher proportions of low frequency (minor allele frequency [MAF] 1–5%) and rare (MAF < 1%) variants, which are the very type of variants of interest. In 660 Alzheimer’s disease cases with earlier onset ages of ≤65, 4 out of 13 (31%) previously-published rare pathogenic and protective mutations in *APP*, *PSEN1*, and *PSEN2* genes were undetected by the default one-pipeline approach but recovered by the multi-pipeline approach.

**Conclusions:**

Identification of the complete variant set from sequencing data is the prerequisite of genetic association analyses. The current analytic practice of calling genetic variants from sequencing data using a single bioinformatics pipeline is no longer adequate with the increasingly large projects. The number and percentage of quality variants that passed quality filters but are missed by the one-pipeline approach rapidly increased with sample size.

**Electronic supplementary material:**

The online version of this article (10.1186/s12859-018-2151-0) contains supplementary material, which is available to authorized users.

## Background

**Fig. 1:**
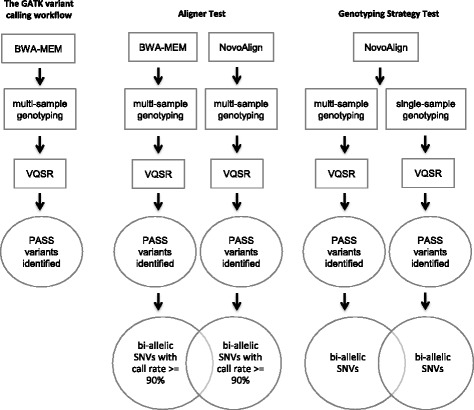
The variant analysis workflow. **a** The default variant calling workflow recommended by GATK (**b**) The workflow to test the benefit of an additional sequence aligner. **c** The workflow to test the benefit of both single-sample and multi-sample-joint genotyping

The identification of genetic risk factors in complex diseases has shifted to rare variants discovery by large sequencing-based studies in search for the substantial missing heritability despite decades of GWAS. Unlike the array-based GWAS, where a pre-defined list of variants are “seeded” on the array surface and called in every sample, variant calling from sequencing data relies on bioinformatics algorithms for variant discovery and quality filtering to obtain a final set of variants for association analyses. The read-to-variant analytic pipelines, such as the most popular Genome Analysis Toolkit (GATK) [1, 2] (Fig. 1a) and other well-accepted analytic workflows [3, 4], have been routinely used for sequencing-based germline variant discovery. However, these workflows were established for much smaller sample sizes and need to be re-examined for today’s increasingly large sample sizes.

A variant calling pipeline typically includes two main steps [5]. First, the reads are mapped to the human reference genome using one single selected alignment tool. Different aligners are designed to optimize the detection of different types of variants while balancing speed, sensitivity, and specificity [6, 7]. Studies have shown that the performances of most aligners are similar [8], and the impact of aligner choice on the consequent variant call is small [9], while the read alignment is computationally expensive and time-consuming. Therefore, the use of any one of several popular alignment tools including Bowtie [10], BWA-MEM [11], and NovoAlign (Novocraft Technologies Sdn Bhd, Selangor, Malaysia) is currently accepted. However, performance analysis of the different aligners has been done on very small numbers of simulated or real sequencing samples and the conclusions may not apply to larger sample sizes. For example, the differences in mapping accuracy between Bowtie, BWA, and NovoAlign are well below 1% using one single simulated sample [7], but the cumulative differences in accuracy may increase significantly if the rare alleles within a larger population of individuals are considered collectively. Consequently, the impact on genotype calls of using one aligner vs. another might be more substantial for sequencing projects with larger sample sizes.

The second step of genetic variant discovery is variant calling, which includes variant identification, variant quality control (QC) and filtering. Currently the GATK best practices analytic guidelines recommend using HaplotypeCaller, followed by multi-sample-joint genotyping (which genotypes a group of samples together) instead of single-sample genotyping (which genotypes variants in individual samples independently without making use of information from other samples) [12, 13]. The benefits of multi-sample-joint genotyping include: (i) greater sensitivity for low-frequency variants due to the ability to call variants at sites where a carrier has low coverage/quality but other samples within sample group have a confident variant at that location, and (ii) greater ability to filter out false positives because the statistical models for variant quality estimation work better with larger amounts of data. However, this practice also calls for a closer examination of variant calling performance with increasingly large sample sizes. The small proportion of variants missed by not performing single sample genotyping on the small numbers of individuals used for most performance evaluation studies is likely negligible. However, the use of large sample sizes may lead to the collective loss of a substantial number of valid but low-frequency variants using the statistical models applied in multi-sample-joint genotyping.

Here, we used the publically available Alzheimer’s Disease Sequencing Project (ADSP) exome dataset [14] which was generated to study AD, a common neurodegenerative disease which usually affects individuals at old age; however, about 10% of patients have onset of symptoms before 65 (early-onset AD; EOAD). Research over the past two decades established that mutations in 3 genes: the amyloid precursor protein gene (*APP*, NM_000484), presenilin 1 gene (*PSEN1*, NM_000021) and presenilin 2 gene (*PSEN2*, NM_000447) can cause autosomal dominant forms of EOAD. Using the ADSP dataset, we discovered that the choices of alignment and variant calling strategies had substantial impact on the number of variants called in a sample size-dependent manner. We further identified a large number of good-quality variants from the ADSP exome data that were missed by the commonly used “best practices” of calling variants by one single pipeline. Our findings revealed relationships between the bioinformatics pipelines employed by the researchers and the discovery of disease variants, and suggested that comparison studies and alternative variant calling strategies may be beneficial for optimal variant discovery from large datasets.

## Methods

### Dataset description

We downloaded Sequence Read Archive (SRA) files of 10,933 ADSP individuals (5787 Alzheimer’s Disease (AD) cases and 5146 controls) from dbGap and converted to FASTQ files using the SRA Toolkit [15].The FASTQ files were processed using the Mayo Clinic GenomeGPS DNA Analysis Pipeline (v3.0.2) (formerly named as TREAT) [16]: reads were aligned to human reference genome GRCh37 using NovoAlign; after local realignment and base quality recalibration, the variants were called using GATK HaplotypeCaller and multi-sample-joint genotyping. The variant quality control (QC) was performed using GATK Variant Quality Score Recalibration (VQSR).

The sample- and population-level QC was performed using an in-house tool kit and PLINK2 [17]. The sample-level QC removed samples not meeting the following criteria: (1) ≥10× coverage for at least 90% of targeted exome regions, and ≥ 40× coverage for at least 30% of targeted regions (26 samples removed); (2) minimum variant call rate of 95% per sample (29 samples removed); (3) average variant Transition/Transversion (Ti/Tv) ratio of least 2.8 (0 sample removed); (4) sample contamination as estimated by FREEMIX statistics > 0.02 [18] (143 samples removed); (5) sex check (gender error is defined as PLINK F estimate < 0.7 for males and > 0.3 for females) (68 samples removed); and (6)APOE genotypes match between the exome data and the sample meta-data (337 samples removed). In addition, the population-level QC removed 146 1st and 2nd degree relatives, 265 non-Caucasian, and 34 samples due to batch effect. Note that some samples failed more than one criterion. We retained a total of 10,033 samples post-QC, which served as the pool from which different sample sizes were selected for this manuscript (the details of sample and population level QC are described in a separate manuscript).

### Testing the benefit of additional sequence aligners

We selected a total of 1952 samples from the 10,033 sample pool for the aligner test, including 660 EOAD cases and 1292 age and gender matched controls. This sample set was part of an on-going EOAD project, and chosen with the limitation of available computational resources for performing read alignments using multiple aligners in mind. We aligned the FASTQ files from these 1952 samples to the human reference genome GRCh37 twice, using BWA-MEM and NovoAlign at default settings, respectively (Fig. 1b). After each alignment, duplicate reads were marked using Picard (v1.119). The BAM files were re-aligned around INDELs using the GATK IndelRealigner and recalibrated using the GATK BaseRecalibrator programs (v3.3–0). After realignment, variant calling and multi-sample-joint genotyping were performed using GATK HaplotypeCaller (v3.3–0) and GenotypeGVCFs (v3.4–46) for the following sample sizes: 50,100, 200, 500, 1000, and 1952. The GATK VQSR was used for variant quality score calculation. Functional annotations of variant sites were performed using ANNOVAR (version 2016Feb01) [19]. For this proof-of-principle study, only bi-allelic single nucleotide variants (SNVs) that received VQSR PASS scores with variant call rate of at least 90% across samples were included in the analyses (Fig. 1b).

### Testing the benefit of both single-sample and multi-sample-joint genotyping

We randomly selected 10,000 samples for the genotyping strategy comparison test. The FASTQ files of the 10,000 samples were aligned to the human reference genome GRCh37 using NovoAlign at default settings. After alignment, duplicate reads were marked using Picard (v1.119). The BAM files were re-aligned around INDELs using the GATK IndelRealigner and recalibrated using the GATK BaseRecalibrator programs, respectively (v3.3–0). Single-sample variant calling and single-sample genotyping were performed using GATK HaplotypeCaller (v3.3–0) and GenotypeGVCFs (v3.4–46) for all 10,000 samples. Joint genotyping was performed for the following sample sizes: 50, 100, 500, 2000, 5000 and 10,000. Functional annotations of variant sites were performed using ANNOVAR (version 2016Feb01). Only bi-allelic SNVs that received VQSR PASS scores were used in the analyses. Minimum variant call rate was not required here because it cannot be properly calculated for single-sample genotyping (Fig. 1c).

### Variant quality assessment

In addition to VQSR, the quality of variants was assessed by the following metrics when applicable: 1) B Allele proportion (BAP), calculated as the percentage of reads supporting the alternative allele; 2) B allele frequency (BAF), calculated as the frequency of the alternative allele in given sample sizes; 3) number of samples with the B allele, calculated when BAF calculation is not applicable; 4) Genotype Quality (GQ) scores provided by VCF files; 5) Depth of sequencing (DP) calculated as the total number of reads at the variant position; 6) average GC content of 100 bases flanking variants of interest; 7) variant distribution across chromosome; and 8) overlap of the variant position with genomic regions with mapping difficulties including the low complexity region (LCR) [20] and segment duplication regions (SDR) [21].

## Results

### Discovery of additional variants by using two different alignment methods

**Fig. 2:**
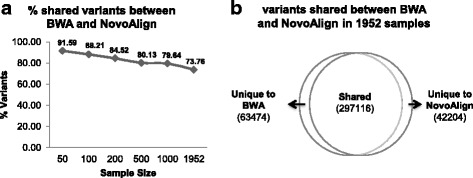
Using two sequence aligners identified overlapping and unique variants. **a** The percentage of overlap in variants identified by BWA-MEM and NovoAlign decreased substantially with increasing sample sizes. **b** Comparison of variants identified by BWA-MEM and NovoAlign in 1952 samples

We compared variants called from BAM alignment files generated by either BWA-MEM or NovoAlign from the ADSP dataset at samples sizes of 50, 100, 200, 500, 1000, and 1952. For each sample size except for the largest, we randomly sampled 5 times and compared the average. We found that the shared variants identified by both BWA-MEM and NovoAlign decreased substantially from 91.59% to 73.76% when sample sizes increased from 50 to 1952 (Fig. 2a). At sample size of 1952, 63,474 variants were uniquely identified by BWA-MEM, accounting for 15.76% of total variants, and 42,204 variants were uniquely identify by NovoAlign, accounting for 10.48% of total variants (Fig. 2b).

### The quality metrics of aligner-specific variants

**Fig. 3:**
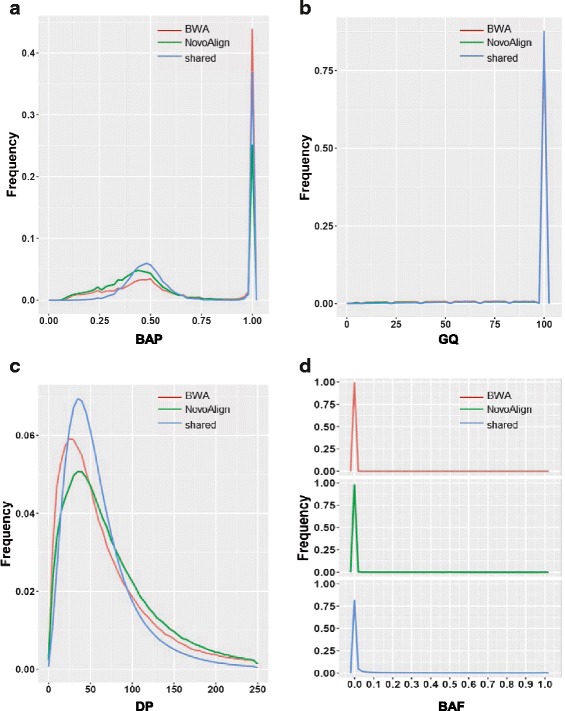
The quality metrics of BWA-unique, Novo-unique and shared variants at sample size 1952. **a** The distribution of BAP in BWA-unique (red), Novo-unique (green) and shared variants (blue). **b** The distribution of GQ in BWA-unique (red), Novo-unique (green) and shared variants (blue). **c** The distribution of DP in BWA-unique (red), Novo-unique (green) and shared variants (blue). **d** The distribution of BAF in BWA-unique (red), Novo-unique (green) and shared variants (blue)

The large discrepancy between the sets of variants identified from alignments by BWA-MEM and NovoAlign in this dataset led us to investigate whether the variants uniquely identified by one aligner but not the other were of reliable quality. We investigated several main variant quality metrics, including B allele proportion (BAP), genotype quality (GQ), read depth (DP), and B allele frequency (BAF), and compared them among the three groups of variants: variants uniquely identified from BWA-MEM alignments(BWA-unique), variants uniquely identified from NovoAlign alignments(Novo-unique), and variants identified from alignments by both aligners (shared). At our largest sample size (*n* = 1952), we found that BWA-unique, Novo-unique and shared variants had similar distributions of BAP, centering at 0.5 and 1 (Fig. 3a), consistent with the characteristics of diploid genomes. The three groups of variants also demonstrated similar distribution of high GQ (Fig. 3b) and DP (Fig. 3c), indicating comparable genotype confidence and depth of coverage. Interestingly, a larger difference among the three groups of variants was shown in BAF (Fig. 3d): 98.99% of BWA-unique and 97.31% of Novo-unique variants had BAF < = 0.5%, higher than that of the shared variants (77.80% variants had BAF < =0.5%), suggesting that more of the aligner-specific variants are rare in the population.

### The genomic location and GC content of the aligner-specific variants

**Fig. 4:**
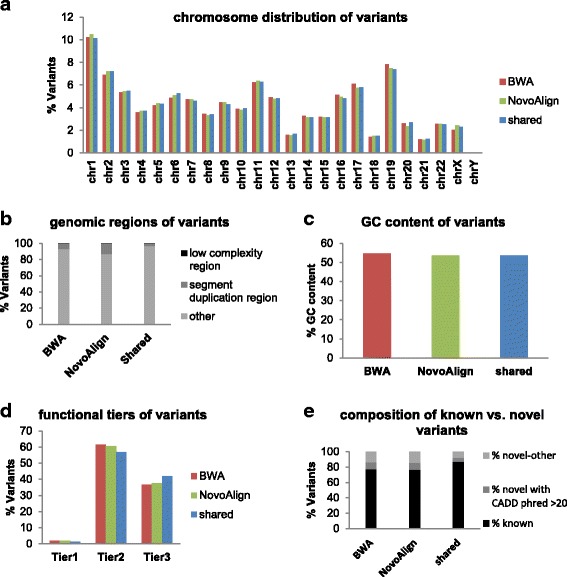
Characteristics of the aligner-specific variants. **a** The chromosome distribution of BWA-unique (red), Novo-unique (green) and shared variants (blue). **b** The percentage of variants in the LCR, SDR and other regions among BWA-unique, Novo-unique, and shared variants. **c** The average GC content of BWA-unique (red), Novo-unique (green) and shared variants (blue). **d** The composition of different functional tiers in BWA-unique (red), Novo-unique (green) and shared variants (blue). Tier 1 includes variants that disrupt the start or stop codon, or cause splicing events; Tier 2 includes variants that cause non-synonymous changes, and Tier3 includes all other types. **e** The composition of known and novel variants in BWA-unique, Novo-unique and shared variants

In order to further characterize the aligner-specific variants, we compared the genomic regions in which the three groups of variants are located. Specifically, exons from genomic regions such as the Low Complexity Region (LCR) and Segment Duplication Regions (SDR) are known to have mapping difficulties, which can be reflected in different calling sensitivities resulting from different aligners. At sample size 1952, we found that BWA-unique, Novo-unique, and shared variants were distributed similarly across chromosomes (Fig. 4a). Less than 0.2% of variants from each variant group were located inside of the LCR. However, compared to shared variants (which had only 2.95% variants located inside of SDR), 6.78% of BWA-unique variants and 12.76% of Novo-unique variants mapped to the SDR (Fig. 4b, Table 1). This result indicates that some unique variants may have been missed by one of the two aligners due to the different ability of alignment algorithms to properly map reads in difficult genomic regions. The fact that a higher percentage of Novo-unique variants are located in the SDR is consistent with previous reports that NovoAlign has better mapping sensitivity [22]. The three variant groups had similar average GC content in flanking regions (Fig. 4c, Additional file 1: Table S1).

**Table 1:** Previously-published pathogenic and protective variants detected in known EOAD genes using the default and alternative approaches

Gene	Protein change	Publication	GnomeAD EUR non-Fin BAF	Called by default pipeline
*APP*	p.V717F	[25]	0	Yes
*APP*	p.I716T	[26]	0	No
*APP*	p.A673T	[27, 28]	0.0003632	No
*PSEN1*	p.A79V	[29, 30]	0.00002369	Yes
*PSEN1*	p.G206A	[31, 32]	0	Yes
*PSEN1*	p.H214Y	[33, 34]	0.000008952	No
*PSEN1*	p.P218L	[35]	0.0000179	Yes
*PSEN1*	p.L262F	[36]	0	Yes
*PSEN1*	p.R269H	[37, 38]	0	Yes
*PSEN1*	p.A396T	[34]	0	Yes
*PSEN2*	p.A85V	[24]	0.000008955	Yes
*PSEN2*	p.L238P	[39]	0.00002687	Yes
*PSEN2*	p.R284G	[40]	0.000008952	No

### Biological relevance of the aligner-specific variants

Because the impact of a genetic variant often relies on its impact on protein function, different levels of research priority are often given to variants with different functional impacts. We therefore categorized variants identified in the 1952 samples into three tiers based on their functional importance: Tier 1 includes variants that disrupt the start or stop codon, or cause splicing events; Tier 2 includes variants that cause amino acid changes (non-synonymous), and Tier 3 includes all other types of SNVs. We annotated BWA-unique, Novo-unique and shared variants using ANNOVAR, and compared the composition of Tier 1, 2, and 3 in the three groups of variants based on the annotation. Our results showed that the tier composition was comparable among the three groups of variants (Fig. 4d, Additional file 2: Table S2).

We next evaluated whether some of the aligner-specific variants have been recorded in public databases. We searched the three groups of variants in the following databases: dbSNP build 147, Exome Sequencing Project (ESP), ClinVar, the 1000 Genomes Project (1000G), The Exome Aggregation Consortium (ExAC), Kaviar Genomic Variant Database (Kaviar), and the Haplotype Reference Consortium (HRC). At a sample size of 1952, 77.53% of the BWA-unique and 76.7% of Novo-unique variants were found in public databases, suggesting that the majority of aligner-specific variants are likely true positives. In addition, 8.85% of BWA-unique, and 9.4% of Novo-unique variants were novel variants that had a Combined Annotation Dependent Depletion (CADD) score (PHRED-like) of at least 20 [23], indicating that these variants are amongst the top 1% of deleterious variants in the human genome and likely biologically relevant (Fig. 4e, Additional file 3: Table S3).

### Single-sample genotyping added variants not identified by joint genotyping

**Fig. 5:**
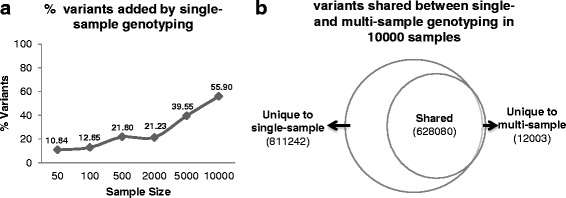
Single-sample genotyping added variants not identified by multi-sample-joint genotyping. **a** single-sample genotyping added increasing percentage of variants with increasing sample size (**b**) comparison of variant call-sets between single-sample genotyping and joint genotyping at sample size of 10,000

In order to assess the contribution of single-sample genotyping, we compared variant call-sets generated by single-sample genotyping and those generated by multi-sample-joint genotyping at sample sizes of 50, 100, 500, 2000, 5000 and 10,000. For each sample size except for the largest, we randomly sampled 5 times and compared the average. We found that the percentage of variants uniquely identified by single-sample genotyping dramatically increased with sample size (Fig. 5a). At a sample size of 10,000, single-sample genotyping added 811,242 variants, accounting for 55.9% of total called variants. Multi-sample-joint genotyping, on the other hand, identified 12,003 variants that were not identified by single-sample genotyping, accounting for 0.83% of total called variants (Fig. 5b).

### The quality metrics of variants uniquely called by single-sample genotyping

**Fig. 6:**
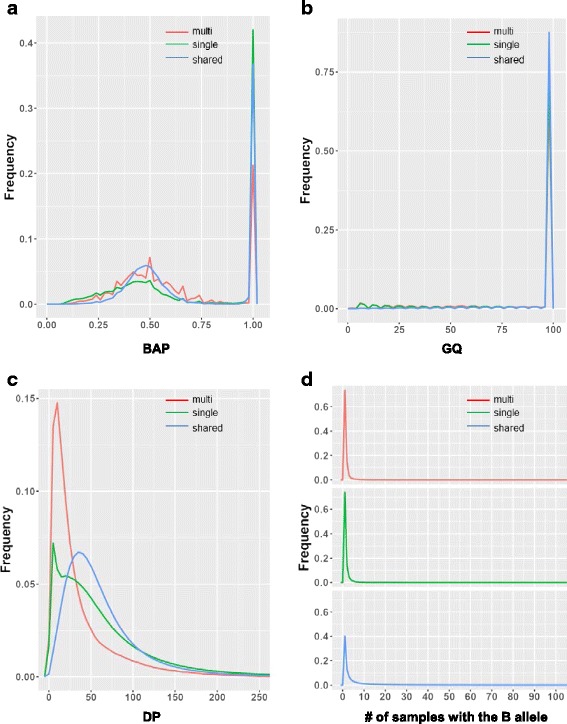
The quality metrics of 3 groups of variants at sample size n10,000. **a** The distribution of BAP in multi-unique (red), single-unique (green) and shared variants (blue). **b** The distribution of GQ in multi-unique (red), single-unique (green) and shared variants (blue). **c**The distribution of DP in multi-unique (red), single-unique (green) and shared variants (blue). **d** The distribution of the number of samples having the B allele in multi-unique (red), single-unique (green) and shared variants (blue)

In order to learn about the quality of the variants uniquely identified by single-sample genotyping, we compared BAP, GQ, DP and the number of samples with the B allele among the three groups of variants: variants uniquely identified by multi-sample-joint genotyping (multi-unique), variants uniquely identified by single-sample genotyping (single-unique), and variants identified by both strategies (shared). We found that the three groups of variants had similar BAP and GQ distribution (Fig. 6a, b). However, compared to shared variants, both multi-unique and single-unique variants showed lower depth of coverage (Fig. 6c), suggesting that variants with lower coverage may be more sensitive to genotyping strategies. Due to the fact that we cannot differentiate between no call and a same-as-reference call in single-sample genotyping, we were unable to properly plot BAF for single-sample genotyping. Instead, we compared the number of samples with the B allele among the three variant groups (Fig. 6d). Interesting, we discovered that 94.5% of single-unique variants were present in less than 5 samples (73.81% were singletons), and 93.4% of multi-unique variants were present in less than 5 samples (73.51% were singletons), higher than that of the shared variants among which 64.5% were present in less than 5 samples (40% singletons), suggesting that single-sample genotyping has higher sensitivity to rare variants.

### The genomic location and GC content of variants uniquely identified by single-sample genotyping

**Fig. 7:**
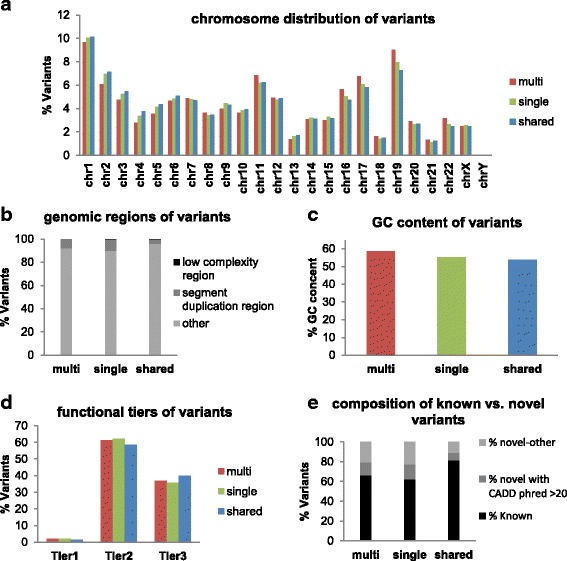
Characteristics of multi-unique, single-unique and shared variants. **a** Chromosome distribution of multi-unique (red), single-unique (green) and shared variants (blue). **b** The percentage of variants in the LCR, SDR and other regions among multi-unique, single-unique and shared variants. **c** The average GC content flanking of multi-unique (red), single-unique (green) and shared variants (blue). **d** The functional tiers in multi-unique (red), single-unique (green) and shared variants (blue). Tier 1 includes variants that disrupt the start or stop codon, or cause splicing events; Tier 2 includes variants that cause non-synonymous changes, and Tier3 includes all other types. **e** The composition of known and novel variants in multi-unique, single-unique and shared variants

We next compared the genomic regions in which the three groups of variants are located. We found that the three groups of variants are distributed similarly across chromosomes (Fig. 7a). The three groups of variants have a similarly low percentage to the aligner-specific variants located inside of the LCR and SDR (Fig. 7b, Additional file 4: Table S4). Interestingly, the average GC content flanking multi-unique variants are slightly higher than single-unique and shared variants (Fig. 7c, Additional file 4: Table S4). These data suggest that variants added by single-sample genotyping have comparable characteristics to the shared variants, and that the some of the multi-unique variants may not have been identified by single-sample genotyping due to the characteristics of the DNA sequence surrounding these variants.

### Biological relevance of variants uniquely identified by single-sample genotyping

As we did for the aligner comparison above, we categorized variants identified in the 10,000 samples into three functional tiers and compared the composition of Tier 1, 2, and 3 among single-unique, multi-unique and shared variants. Our results showed that the tier composition was similar among the three groups (Fig. 7d, Additional file 5: Table S5). In addition, we compared variants from each group to public databases including dbSNP build 147, ESP, ClinVar, 1000G, ExAC, Kaviar, and HRC. We found that 61.98% of single-unique variants were recorded in public databases, suggesting that at least this proportion of these variants are likely true positives (Fig. 7e, Additional file 6: Table S6). Also, 15.36% of single-unique variants identified are novel variants that had a CADD score (PHRED-like) of at least 20, indicating that these variants were amongst the top 1% of deleterious variants in the human genome. Again, this strongly indicates biological relevance of a substantial proportion of the single-unique variants detected.

### Discovery of additional pathogenic and protective EOAD variants using multiple aligners and genotyping strategies

To demonstrate the benefit of using multiple aligners together with multiple genotyping strategies, especially in the context of rare variants, we evaluated whether additional pathogenic or protective mutations could be identified in the 3 established autosomal dominant EOAD genes (APP, PSEN1 and PSEN2), by using these alternative approaches. In our EOAD patient cohort, the default strategy (BWA-MEM followed by multi-sample-joint genotyping) identified nine known pathogenic mutations in the three genes: one in *APP*, six in *PSEN1*, and two in *PSEN2*. One of the *PSEN2* mutations, p.A85V, was found in an 89-year old control individual; however, this mutation is known for its large clinical variability and late age at onset [24]. Importantly, by adding NovoAlign as an alternative aligner and by performing single-sample genotyping in addition to multi-sample-joint genotyping, we identified four additional previously-published pathogenic or protective variants: two in *APP* (p.I716T and p.A673T), one in *PSEN1* (p.H214Y), and one in *PSEN2* (p.R284G) (Table 1). Overall, our alternative strategies identified 4 out of 13 (31%) previously-published rare pathogenic or protective mutations in *APP*, *PSEN1*, and *PSEN2* genes that were undetected by the default variant calling approach, which are likely true positives. The full list of variants identified in *APP*, *PSEN1* and *PSEN2* by each workflow is shown in Additional file 7: Table S7.

## Discussion

### The multi-pipeline approach can rescue a substantial amount of variants with potential biological significance

Our aligner comparison between BWA-MEM and NovoAlign identified a large number of variants that would have been missed by using either one of the two aligners compared. In a cohort of 1952 individuals, using BWA-MEM alone would have led to the identification of 42,204 fewer variants, or 10.48% fewer total variants, and using NovoAlign alone would have missed 63,474, or 15.76% of total variants. The two aligners were chosen among other top aligners in this study because they were shown to have good balance between speed and alignment accuracy [6]. Read alignment is the most computationally expensive step, and using these two aligners we were able to demonstrate the necessity of aligning reads using multiple aligners and its impact in identifying missing variants. It is likely that including additional aligners will rescue even more variants in our cohort since different aligners have different preferences that may favor different types of variants.

Similarly, by adding single-sample genotyping on the ADSP WES dataset, we have identified 55.9% additional variants at sample size of 10,000. Furthermore, we showed that a large percentage of the recovered variants had low frequencies in the population, and thus may be extremely valuable in rare variant studies. In experiments with large populations, a variant that only exists in one or a few individuals (private variants) are more likely to be missed by multi-sample-joint genotyping because the reads supporting the alternative allele may be deemed statistically insignificant in the context of thousands of samples, while single-sample genotyping has better sensitivity to such variants. In circumstances where using multiple aligners is impractical due to limitations in financial or computational resources, single-sample genotyping provides a time- and cost-effective alternative to gather more complete variant call-sets.

### Quality and reliability of rescued variants

Because the ADSP dataset is a public dataset, we do not have access to the DNA samples to validate in-lab the variants we identified, which would have been especially informative for the recovered variants identified by our multi-pipeline approach. However, by requiring all rescued variants to pass VQSR, investigating various additional variant quality measures, distribution across the genome, local GC content, overlap with public databases and previous publication, we found that the recovered variants had comparable characteristics to the ones jointly identified by using both aligners or by using both genotyping strategies. What’s more, the majority of recovered variants are known variants in the human population, which gave us confidence that a large number of them are likely true positives. However, we are aware that using multiple approaches inevitably introduces more noises into the final variant call-sets, and that not all recovered variants are true positives even if they demonstrate all normal characteristics. We therefore strongly recommend that researchers take caution when using multiple pipelines for variant discovery, and that additional filtering based on other statistical models or prior biological knowledge may be necessary to control false discovery. At the same time, the assumption that the variants called uniquely/exclusively by a single pipeline are of lesser quality is not sustained by evidence. In fact, all aligners and variant callers have their own biases; therefore the variant call sets by different pipelines complement each other in theory. In experiments with large sample sizes designed to capture rare variants, even a small percentage of missed variants can result in hundreds and thousands of missed opportunities to identify meaningful disease-related genes. The multi-pipeline approach warrants a more complete variant call-set, which is extremely valuable for large scale WES experiments in search of rare variants.

### The unprecedented increase of data volume requires more tool- and parameter- testing to achieve optimal variant discovery outcome

The rapidly increasing sample sizes of sequencing-based genetic studies of complex disease pose new challenges to the read-to-variant analytics. The identification of a complete set of genomic variants, common and rare, is of paramount importance before association analyses.

While our study demonstrated the benefit of using multiple variant calling pipelines for WES data, it is important to note that other types of NGS data may have optimal outcomes from different combinations of tools and parametric settings to those described here, contingent on sample sizes. Comparison studies of multiple variant-calling methods on datasets from whole genome sequencing, whole transcriptome sequencing, and targeted sequencing are now necessary.

## Conclusions

After decades of genetic research, there are still substantial amount of missing heritability to be identified in complex disease such as the Alzheimer’s. Our study strongly suggested that limitations from current bioinformatics practices might be one of the culprits. Using the ADSP exome data, and by comparing multiple aligners and genotyping strategies, our study showed that today’s common analytic practice of using a single read-to-variant pipeline missed substantial percentage of good quality variants, including previously published pathogenic and protective rare AD variants, in a sample size dependent manner (more loss in larger cohorts). Furthermore, the missed variants are disproportionally of low and rare frequencies, which are the variants of interest for all large sequencing projects. A case study of 660 EOAD patients from ADSP showed that current default pipeline missed 4 out of 13 (31%) of previously published rare pathogenic and protective mutations in three genes known to associate with the disease. Our results support the utilization of multiple analytic approaches in search of rare genetic risk factors in large exome sequencing projects.

## Additional files

Additional file 1: **Table S1.** The genomic location and GC content of BWA-unique, Novo-unique and shared variants. (DOCX 14 kb)

Additional file 2: **Table S2.** The composition of Tier 1, 2 and 3 variants in BWA-unique, Novo-unique and shared variants. (DOCX 13 kb)

Additional file 3: **Table S3.** The percentage of known and novel variants in BWA-unique, Novo-unique and shared variants. (DOCX 13 kb)

Additional file 4: **Table S4.** The genomic location and GC content of multi-unique, single-unique and shared variants. (DOCX 14 kb)

Additional file 5: **Table S5.** The composition of Tier 1, 2 and 3 variants in multi-unique, single-unique and shared variants. (DOCX 14 kb)

Additional file 6: **Table S6.** The percentage of known and novel variants in multi-unique, single-unique and shared variants. (DOCX 13 kb)

Additional file 7: **Table S7.** The full list of variants identified in *APP*, *PSEN1* and *PSEN2* by each workflow. (XLSX 14 kb)

## Abbreviations

1000G: the 1000 Genomes Project
AD: Alzheimer’s disease
ADSP: the Alzheimer’s Disease Sequencing Project
*APP*: amyloid precursor protein
BAF: B allele frequency
BAP: B allele proportion
CADD: Combined Annotation Dependent Depletion
DP: read depth
EOAD: early onset Alzheimer’s disease
ESP: the Exome Sequencing Project
ExAC: the Exome Aggregation Consortium
GATK: the Genome Analysis Toolkit
GQ: genotype quality
GWAS: genome-wide association studies
HRC: the Haplotype Reference Consortium
Kaviar: Kaviar Genomic Variant Database
LCR: Low Complexity Region
MAF: minor allele frequency
*PSEN1*: presenilin 1
*PSEN2*: presenilin 2
QC: quality control
SDR: Segment Duplication Regions
SNV: single nucleotide variant
Ti/Tv: Transition/Transversion
VQSR: Variant Quality Score Recalibration
WES: whole exome sequencing
WGS: whole genome sequencing
