# Supplementary material for: Identification of missing variants by combining multiple analytic pipelines
Source: BMC Bioinformatics. 2018 Apr 16;19:139. doi: 10.1186/s12859-018-2151-0 (PMC5902939; doi:10.1186/s12859-018-2151-0)
Supplement: Supplementary file 1 — Table S1. The genomic location and GC content of BWA-unique, Novo-unique and shared variants. (DOCX 14 kb) [file 12859_2018_2151_MOESM1_ESM.docx]

Table S1. The genomic location and GC content of BWA-unique, Novo-unique and shared variants.

|  | **BWA-unique** | **Novo-unique** | | **Shared** | | |  |
| --- | --- | --- | --- | --- | --- | --- | --- |
| **% in low complexity region** | | 0.15 | 0.14 | | | | 0.11 |
| **% in segment duplication region** | | 6.78 | 12.76 | | | 2.95 | |
| **% in other** | | 93.07 | 87.1 | 96.94 | | | |
| **% GC content** | | 54.64 | 53.60 | 53.64 | | | |
