# Supplementary material for: Identification of missing variants by combining multiple analytic pipelines
Source: BMC Bioinformatics. 2018 Apr 16;19:139. doi: 10.1186/s12859-018-2151-0 (PMC5902939; doi:10.1186/s12859-018-2151-0)
Supplement: Supplementary file 2 — Table S2. The composition of Tier 1, 2 and 3 variants in BWA-unique, Novo-unique and shared variants. (DOCX 13 kb) [file 12859_2018_2151_MOESM2_ESM.docx]

Table S2. The composition of Tier 1, 2 and 3 variants in BWA-unique, Novo-unique and shared variants.

|  | **BWA-unique** | **Novo-unique** | **Shared** |
| --- | --- | --- | --- |
| **% Tier1** | 1.83 | 1.79 | 1.31 |
| **% Tier2** | 61.43 | 60.45 | 56.73 |
| **% Tier3** | 36.73 | 37.76 | 41.96 |

Tier 1 includes variants that disrupt the start or stop codon, or cause splicing events; Tier 2 includes variants that cause non-synonymous changes, and Tier3 includes all other types of SNVs.
