# Supplementary material for: Identification of missing variants by combining multiple analytic pipelines
Source: BMC Bioinformatics. 2018 Apr 16;19:139. doi: 10.1186/s12859-018-2151-0 (PMC5902939; doi:10.1186/s12859-018-2151-0)
Supplement: Supplementary file 3 — Table S3. The percentage of known and novel variants in BWA-unique, Novo-unique and shared variants. (DOCX 13 kb) [file 12859_2018_2151_MOESM3_ESM.docx]

Table S3. The percentage of known and novel variants in BWA-unique, Novo-unique and shared variants.

|  | **BWA-unique** | **Novo-unique** | **Shared** |
| --- | --- | --- | --- |
| **% known** | 77.53 | 76.7 | 87.34 |
| **% novel- CADD phred >=20** | 8.85 | 9.4 | 5.14 |
| **% novel-other** | 13.62 | 13.9 | 7.52 |
