# Supplementary material for: Identification of missing variants by combining multiple analytic pipelines
Source: BMC Bioinformatics. 2018 Apr 16;19:139. doi: 10.1186/s12859-018-2151-0 (PMC5902939; doi:10.1186/s12859-018-2151-0)
Supplement: Supplementary file 4 — Table S4. The genomic location and GC content of multi-unique, single-unique and shared variants. (DOCX 14 kb) [file 12859_2018_2151_MOESM4_ESM.docx]

Table S4. The genomic location and GC content of multi-unique, single-unique and shared variants.

|  | **multi-unique** | **single-unique** | | | **shared** | |  |
| --- | --- | --- | --- | --- | --- | --- | --- |
| **% in low complexity region** | | 0.003 | 0.34 | | | | 0.11 |
| **% in segment duplication region** | | 7.98 | 9.95 | 4.17 | | | |
| **% in other** | | 92.02 | 89.71 | | 95.72 | | |
| **% GC content** | | 58.70 | 55.24 | | 53.70 | | |
