# Supplementary material for: Identification of missing variants by combining multiple analytic pipelines
Source: BMC Bioinformatics. 2018 Apr 16;19:139. doi: 10.1186/s12859-018-2151-0 (PMC5902939; doi:10.1186/s12859-018-2151-0)
Supplement: Supplementary file 5 — Table S5. The composition of Tier 1, 2 and 3 variants in multi-unique, single-unique and shared variants. (DOCX 14 kb) [file 12859_2018_2151_MOESM5_ESM.docx]

Table S5. The composition of Tier 1, 2 and 3 variants in multi-unique, single-unique and shared variants.

|  | **% multi-unique** | **% single-unique** | **% shared** |
| --- | --- | --- | --- |
| **% Tier1** | 1.98 | 2.12 | 1.49 |
| **% Tier2** | 61.18 | 62.06 | 58.47 |
| **% Tier3** | 36.84 | 35.82 | 40.05 |

Tier 1 includes variants that disrupt the start or stop codon, or cause splicing events; Tier 2 includes variants that cause non-synonymous changes, and Tier3 includes all other types.
