# Supplementary material for: Identification of missing variants by combining multiple analytic pipelines
Source: BMC Bioinformatics. 2018 Apr 16;19:139. doi: 10.1186/s12859-018-2151-0 (PMC5902939; doi:10.1186/s12859-018-2151-0)
Supplement: Supplementary file 6 — Table S6. The percentage of known and novel variants in multi-unique, single-unique and shared variants. (DOCX 13 kb) [file 12859_2018_2151_MOESM6_ESM.docx]

Table S6. The percentage of known and novel variants in multi-unique, single-unique and shared variants.

|  | **multi-unique** | **single-unique** | **shared** |
| --- | --- | --- | --- |
| **% known** | 66.16 | 61.98 | 81.35 |
| **% novel-CADD phred >=20** | 13.37 | 15.36 | 7.74 |
| **% novel-other** | 20.47 | 22.65 | 11.18 |
